# Parkinson's disease and posture: evaluation with biometrical holistic of human body device: a pilot study

**DOI:** 10.3389/fsurg.2024.1413806

**Published:** 2024-11-13

**Authors:** Gianpaolo Ronconi, Paola E. Ferrara, Sefora Codazza, Dario M. Gatto, Fabiana La Cagnina, Daniele Coraci, Maurizio Panunzio, Mariantonietta Ariani, Giorgio Ferriero

**Affiliations:** ^1^ Department of Geriatrics, Orthopedics and Rheumatology, Università Cattolica del Sacro Cuore, Rome, Italy; ^2^ Fondazione Policlinico Universitario “Agostino Gemelli” IRCCS, Rome, Italy; ^3^ Local Health Company, ASL Frosinone, Rome, Italy; ^4^ Department of Neuroscience, Sense Organs and Thorax, Catholic University of the Sacred Heart, Rome, Italy; ^5^ Physical and Rehabilitation Medicine, University of Rome Tor Vergata, Rome, Italy; ^6^ Department of Neuroscience, Section of Rehabilitation, University of Padova, Padua, Italy; ^7^ Responsible Research Hospital, Campobasso, Italy; ^8^ Unit of Physical and Rehabilitation Medicine, Istituti Clinici Scientifici Maugeri IRCCS, Tradate, Varese, Italy; ^9^ Department of Biotechnology and Life Sciences, University of Insubria, Varese, Italy

**Keywords:** Parkinson’s disease, scoliosis, postural imbalance, neurophysiology, rehabilitation

## Abstract

**Background:**

Patients with Parkinson's disease (PD) commonly develop severe spinal deformity. The etiology of Parkinson's spinal deformity is not completely understood and in most cases is likely due to multiple factors. These include dystonia and focal myopathy. Clinical, neurophysiological, and radiological data must be considered to monitor the pathology and the effects of rehabilitation. The aim of this pilot study is to evaluate spine alignment with a surface topography analysis of Parkinson's patients with Biometrical Holistic of Human Body (BHOHB®) and to compare results with their x-rays spine standard as already done for adolescent scoliosis.

**Methods:**

32 adult patients affected by Parkinson disease, have been evaluated with BHOHB ®. The correspondence of the Cobb angles were evaluated using the BHOHB device and with standard spinal x-rays.

**Results:**

A total of 32 patients were enrolled. The mean age was 67.45 years. In this pilot study the measurement of the correlations between the radiological and BHOHB® Cobb angles of the patients were excellent.

**Conclusion:**

This preliminary result supports the use of BHOHB® as a device useful to monitor and measure posture in Parkinson's. This needs to be evaluated on a larger sample and over time. Keywords: Parkinson's disease, scoliosis, postural imbalance, neurophysiology.

## Introduction

Postural spinal abnormalities can be observed in over 20% of patients with Parkinson's disease (PD) during the disease course (1). Patients with Parkinson's disease (PD) commonly develop severe spinal deformity, including scoliosis, anterocollis, camptocormia, and Pisa syndrome. The etiology is due to several causes including alteration of the visual vertical perception and elaboration of basal-ganglia networks, a vestibular system imbalance, peripheral, myopathic muscles changes with abnormalities in axial muscles strength and a dystonic phenomenon due to an altered central integration of proprioceptive signals (2). This becomes particularly evident in more advanced stages of the disease where striking sagittal or coronal plane spinal deviations occur and are further worsened by the process of aging. Postural and radiological spinal data must be considered together with neurophysiological and clinical data to evaluate the evolution of the pathology and the effects of rehabilitation. In the second consensus statement on the diagnosis of MSA and the MDS criteria for diagnosis of MSA, postural deformities were considered as supporting features (3). Standard Spinal x-rays should be considered in the evaluation of Parkinson's patients in association with disability scale and neurophysiological indicators (4). Non-radiological tools for postural assessment as surface topography analysis have been already proposed in literature, with different results regarding the validity and reliability, due to the heterogeneity and poor quality of the studies. Global sagittal balance, describing the vertical alignment of the spine is studied with six non-radiographic methodologies: biophotogrammetry, plumbline, surface topography, infra-red motion analysis, spinal mouse and ultrasound (5). Authors observed that reliability ranged from moderate for spinal mouse to very high for surface topography (6). Surface topography (ST) is applied in the surveillance of scoliosis, with rasterstereography and the Formetric ST System, minimizing radiation exposure in longitudinal care of patients for monitoring disease progression (7). Biometrical Holistic of Human Body (BHOHB-SpinalMeter®) is a surface topography analysis useful for the diagnosis and monitoring scoliosis in adolescents (8). The aim of this pilot study is to evaluate spine alignment of Parkinson's patients with Biometrical Holistic of Human Body (BHOHB-SpinalMeter®) and to measure the correspondence of BHOHB® data with their standard spinal x-ray report.

## Materials and methods

For this non-profit observational interventional study with medical device, 42 consecutive patients with idiopathic PD that presented a scoliotic posture were recruited from the Physical and Rehabilitation outpatient clinic of the Agostino Gemelli University Hospital of Rome between May and October 2022. Eligibility criteria The inclusion criteria were a diagnosis of PD according to the criteria of the Brain Bank of London; Hoehn and Yahr stage II-III; absence of cognitive impairment (MMSE > 24/30); effective pharmacological control of the pathology; acceptance and signature of informed consent. Patients able of maintaining an upright position without the support of aids or orthoses, The exclusion criteria comprised: a diagnosis of atypical Parkinsonism; presence of a clinically diagnosed Pisa syndrome, poor pharmacological compensation of the disease; diagnosis of other neurological, neuromuscular diseases or osteo-articular pathologies; visual impairment or vestibular disorders. Medical examination Patients that met the inclusion criteria underwent a medical examination during which anamnestic data were collected regarding the age, weight, height, body mass index (BMI), disease duration and current PD treatment including daily dose of Levodopa. All the patients were examined in the morning during the “ON” pharmacological phase.

### Radiographic evaluation

Each patient underwent a standard whole-spine x-ray in two planes (antero-posterior and lateral) in orthostatism. A senior radiologist evaluated the radiological images for the presence of spinal scoliosis and other deformities. To avoid misinterpretation with Pisa syndrome, which is a reversible lateral bending of the trunk. Scoliosis was defined as the presence of a radiographic Cobb's angle of at least 10° on the coronal plane, with or without vertebral rotation, that is not corrected by passive movement or supine position. The curve was classified according to the location of its apex (most lateral vertebra) and its extremities (most peripheral upper and lower vertebrae), the direction of the convexity (right or left) and the curvature range (broad or narrow). The presence of other pathological findings in the coronal (e.g., compensation curve) or sagittal plane (e.g., kyphosis, lordosis, listhesis, etc.) was also reported when present.

Biometrical Holistic of Human Body (BHOHB-SpinalMeter®) evaluation Patients were examined clinically and with BHOHB®.

**Figure 1:**
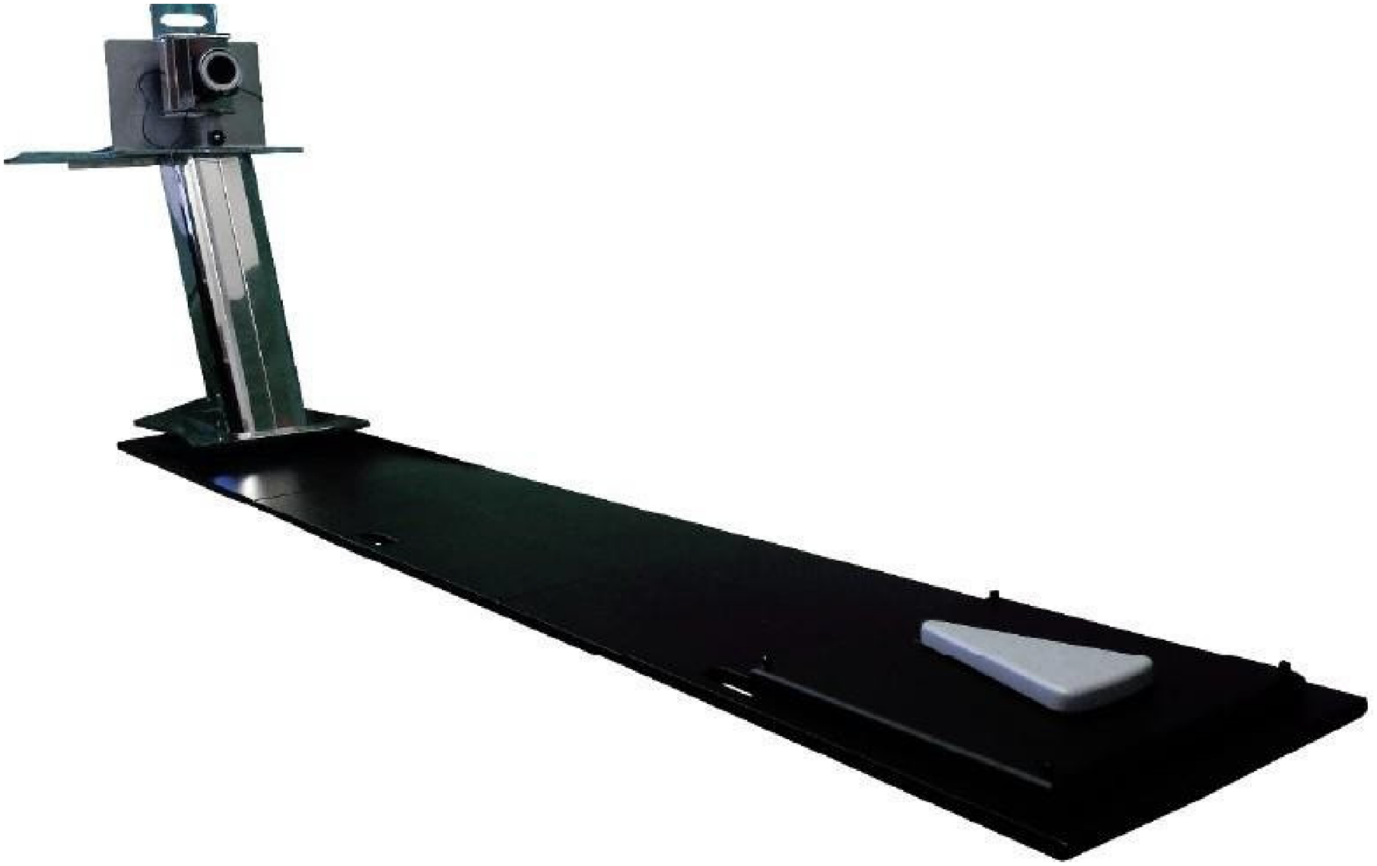
The set points can be palpated on the patient's body as follows: 1. C7 vertebral spinous process. 2. Left acromion. 3. Right acromion. 4. Left inferior scapulae angle. 5. Right inferior scapulae angle. 6. Interscapular vertebral spinous process located midway between the left and right inferior scapular angles. 7. Vertebral spinous process located midway between points 1 and 6. 8. Left posterior superior iliac spine. 9. Right posterior superior iliac spine. 10. Pelvic shelf (usually L5). 11. Vertebral spinous process located midway between points 6 and 10.

**Figure 2:**
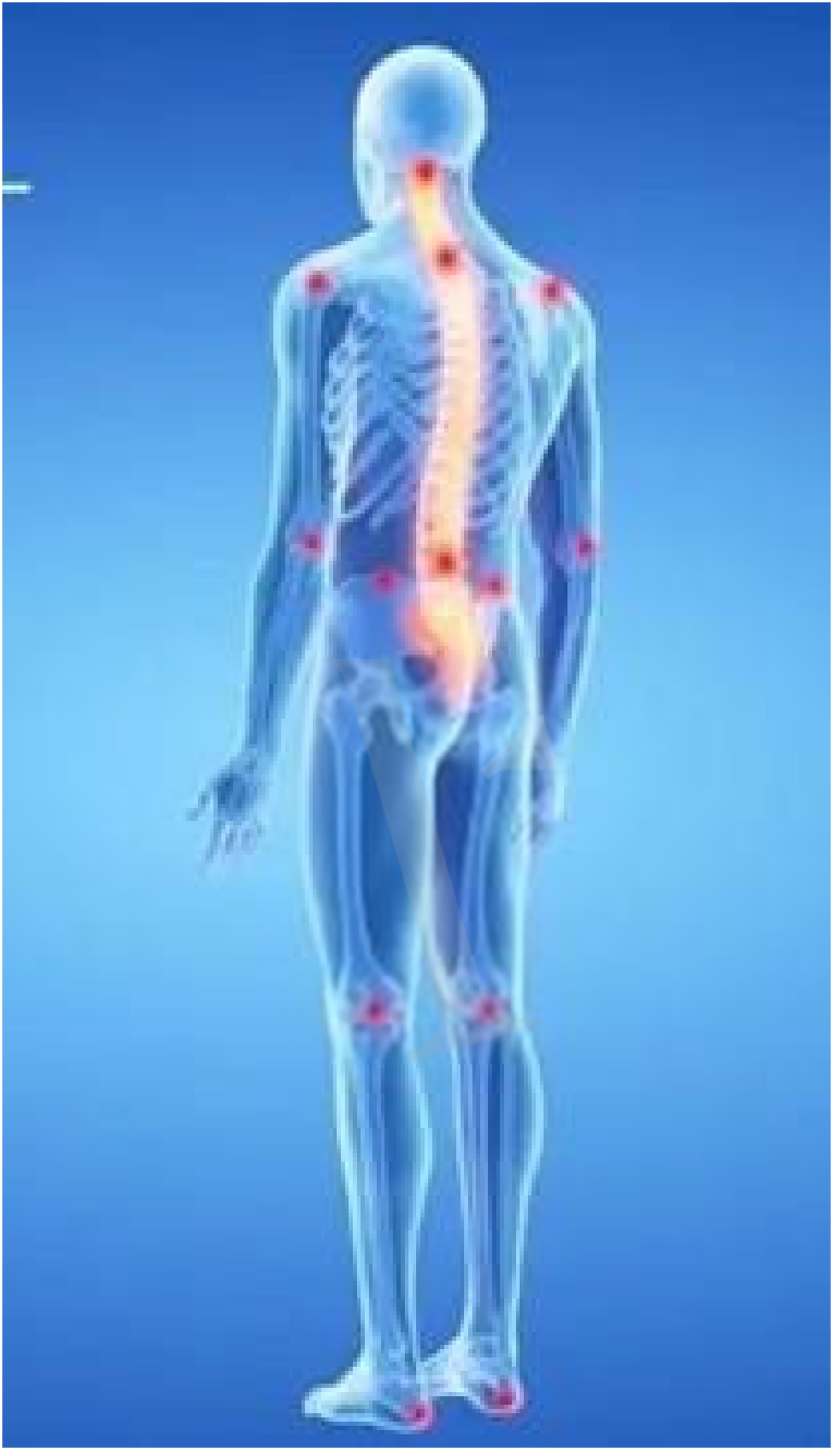
Set point positions.

First, the analysis involved positioning the patient on a special platform (Figure 1) after placing 11 markers in predefined spots on the patient's back (Figure 2).

**Figure 3:**
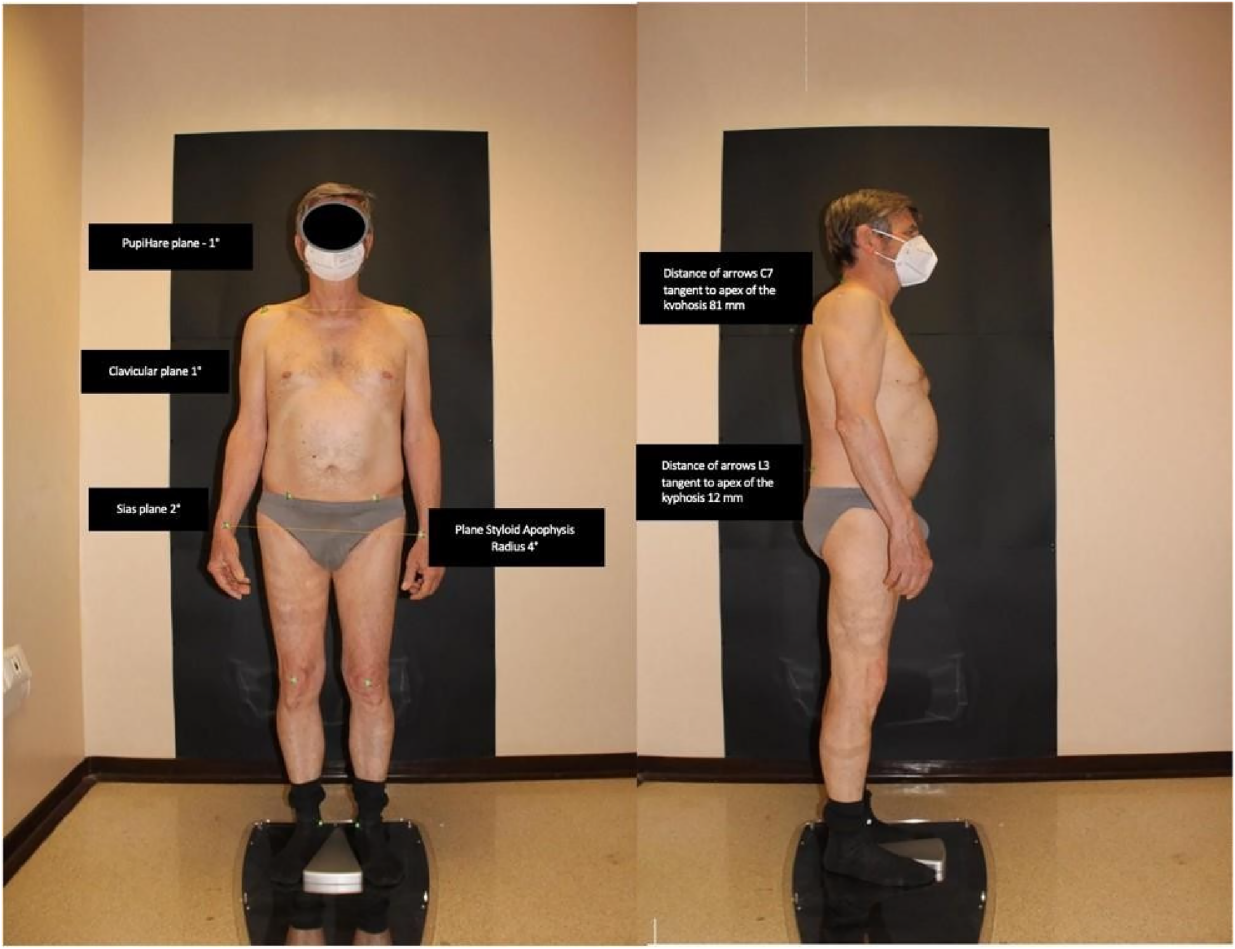
The patient in standard anterior and lateral positions.

The BHOHB device then took two back photos for each exam: the first while the patient was standing (Figure 3).

**Figure 4:**
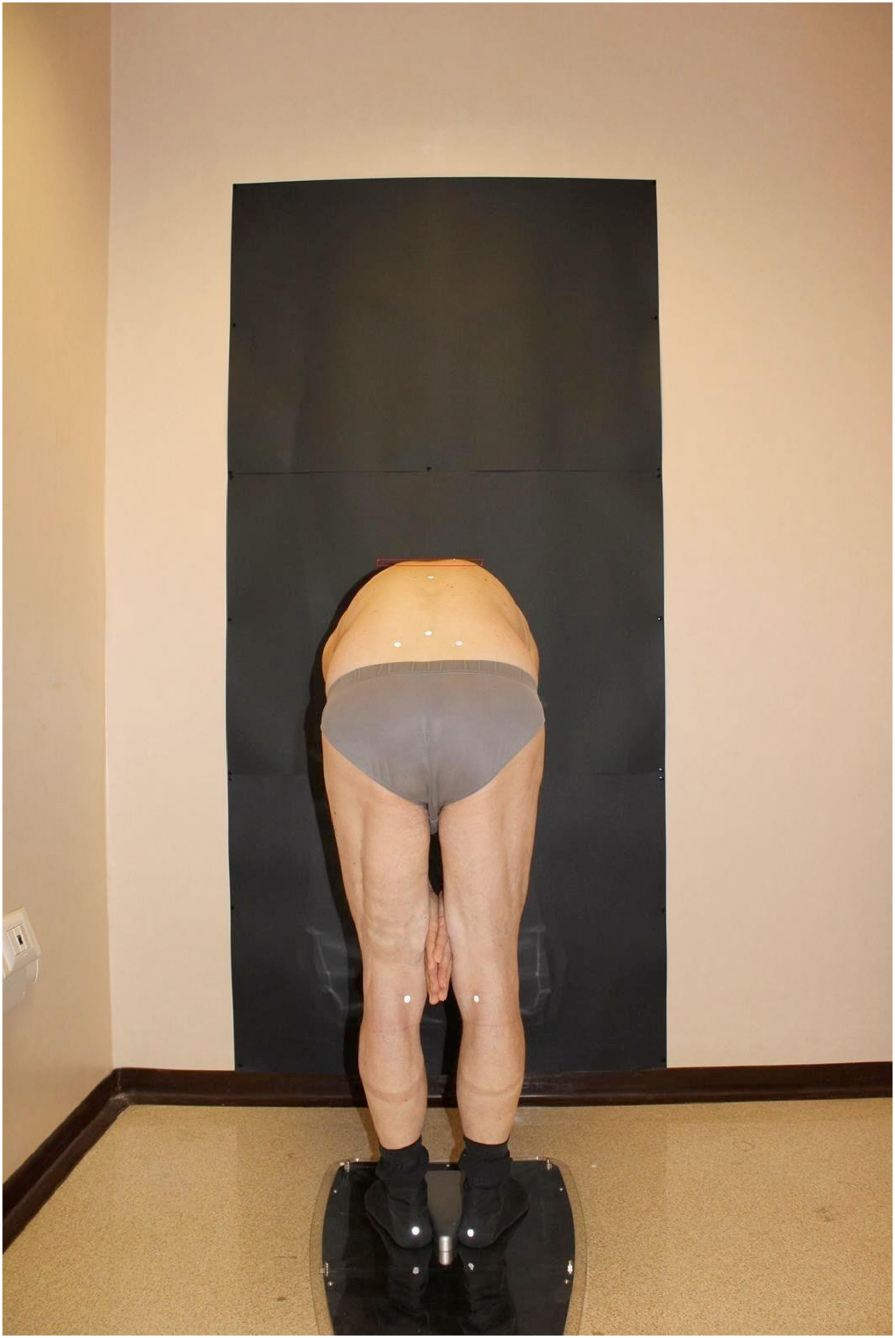
The prominence in an Adams test.

**Figure 5:**
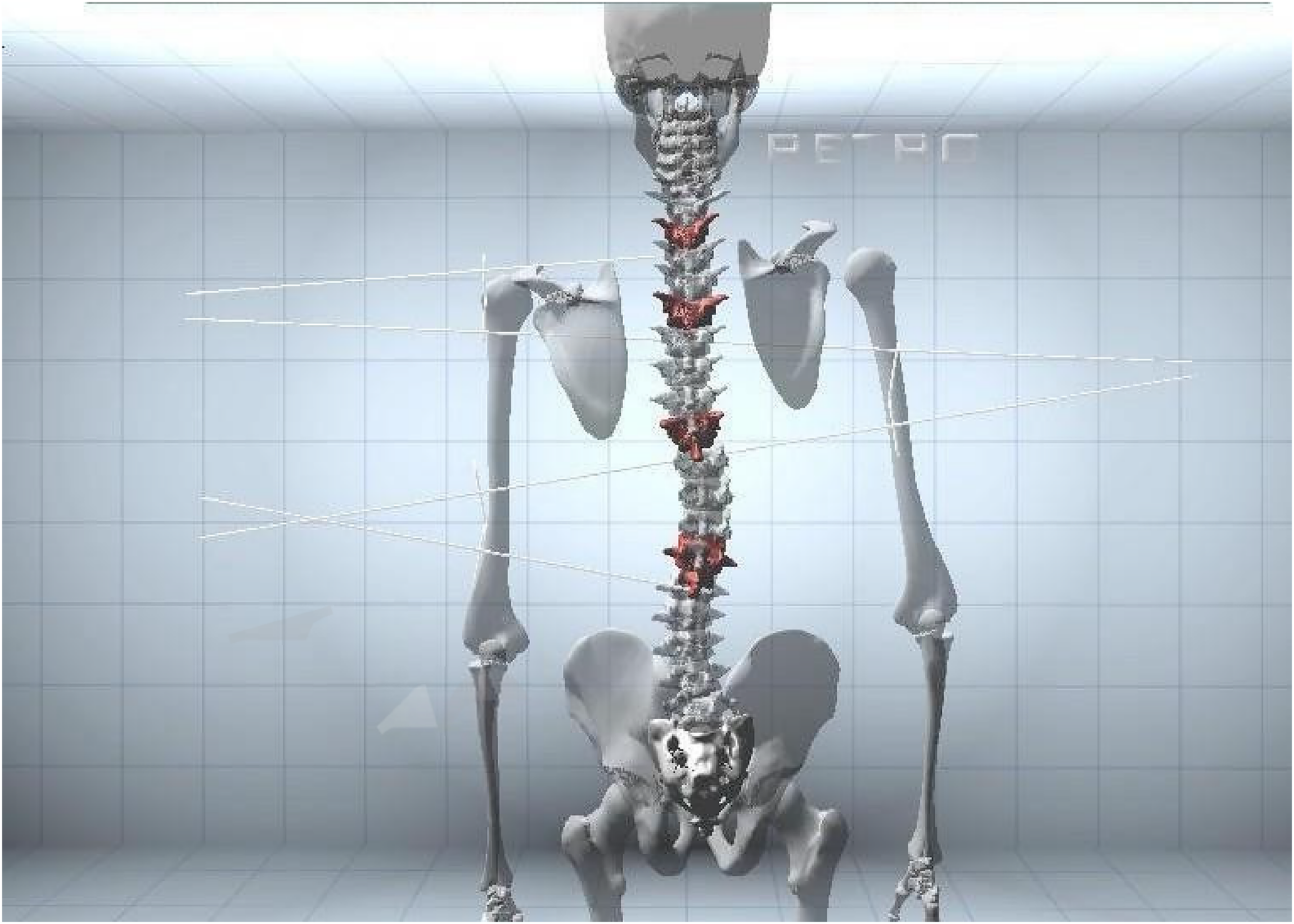
Three-dimensional BHOHB spine reconstruction.

And the second with the patient in the Adams test position (Figure 4). Finally, the BHOHB software provided a 3D reconstruction of the spine (Figure 5). The AP x-ray view was used to determine the patient's curve magnitude (Cobb's method), and the end vertebrae were preselected to minimize interobserver error.

BHOHB technologies are certified by the Ministry of Health and have a European (EP 3225155 A1; application number: 08425006.7) and worldwide patent (PCT/EP2019/082111).

The study was carried out according to the Declaration of Helsinki and the protocol was approved by the Ethics Committee of the Policlinico Gemelli Foundation (UCSC protocol N 0027687/21 30/07/2021). All patients provided their informed consent prior to inclusion in the study.

### Statistical analysis

The validity of the BHOHB technology was estimated using the Bland–Altman method and the Pearson correlation coefficient. Study results (Cobb angle value) were expressed as average and standard deviations; values obtained with our two different methods (x-ray and BHOHB) were compared using Student's *t*-test for paired data.

The correlation between the BHOHB and radiography measurements was expressed by the Pearson correlation coefficient.

In both cases, the correlation coefficient value was evaluated as follows:

— 0.25–0.50: poor correlation.
— 0.50–0.75: moderate to good correlation.
— 0.75–1.00: very good to excellent correlation.

## Results

A total of 42 patients were enrolled. The average age was 67.45 years. 10 patients who did not meet the criteria for performing the BHOHB examination were excluded. From the comparison between the measurement of the radiological Cobb angle (from standard x-ray in orthostatism, on a single plate in the anteroposterior projection) of the patient with that evaluated with BHOHB SPINAL-METER ® The correlations between the measurements carried out with the BHOHB and the Radiograph showed an excellent r: Operator x-ray r = 0.984. Furthermore, examination of the corresponding Bland and Altman graph shows excellent agreement between the two measurements. In fact, the points, which represent the differences between the measurements, fall entirely within the 95% confidence band of the differences in the means.

**Figure 6:**
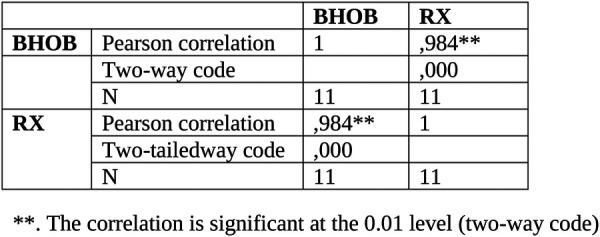
Correlation between Bhohb and x-ray.

Correlations between BHOHB measurements and Radiography showed an excellent r: Operator x-Ray r = 0.984 (Figure 6).

**Figure 7:**
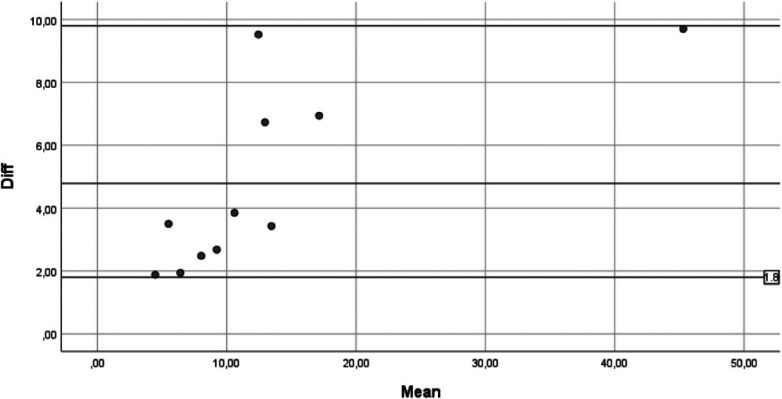
Differences between the measures.

Furthermore, examination of the correspondence graph by Bland and Altman shows very good agreement between the two measurements. In fact, the points, which represent the differences between the measures, fall entirely within the 95% confidence band of the differences in the means (Figure 7).

## Discussion

Our study evaluated spinal alignment using surface topography analysis in Parkinson's disease patients with the Biometrical Holistic of Human Body (BHOHB®) system and compared the results with their standard spinal x-ray reports. The application of standard spinal x-ray for visualizing spinal abnormalities is becoming more popular. This approach proves particularly valuable for monitoring individuals with scoliosis and the correspondence of the Cobb angles measures obtained as it avoids exposure to radiation. In this article we introduced a novel technique: Biometrical Holistic of Human Body (BHOHB®). BHOHB is a medical device used for postural and spinal assessment with a surface topography technology without exposure to x-rays and with savings of economic resources comparing to x-ray. The surface topography (ST) is utilised in the monitoring of scoliosis, reducing radiation exposure in the ongoing care of patients to track disease progression longitudinally (7). Numerous investigations demonstrated that surface topography (ST) exhibited notable consistency with high repeatability along with strong intraobserver and interobserver agreement. The association between ST parameters and the radiographic Cobb angle varied from moderate to high (9, 10). Biometrical Holistic of Human Body (BHOHB-SpinalMeter®) is a surface mapping analysis useful for diagnosing and monitoring scoliosis even in adolescents (8). Another crucial aspect in screening for idiopathic scoliosis is the assessment of prominence. Prominence serves as the most apparent clinical indicator of scoliosis and holds significance as a prognostic factor concerning curve progression. Nevertheless, previous studies have raised doubts regarding the correlation between clinical deformity and the severity of the curve. Recent literature has indicated that a hump measured using a humpmeter becomes significant at a threshold of 5 mm. Additionally, a trunk rotation angle of ≥7° for thoracic and right convex curves and ≥6° for thoracolumbar, lumbar, and left convex curves is considered a reliable criterion for identifying patients with Cobb angles of 25° or greater. This criterion helps in reducing the necessity for spinal radiography (11). Nevertheless, the outcomes of the investigation enable us to assert that through surface mapping we can assess two parameters: the prominence and the Cobb angles. Making it advisable to employ this method before resorting to x-rays for the patient. This dual assessment permits an evaluation not only of the curvature but also of rotation, a critical aspect considering scoliosis as a three-dimensional deformity. Consequently, this facilitates a comprehensive evaluation and notably diminishes the requirement for unnecessary radiographic examinations. The results of our study demonstrate the efficacy of utilizing BHOHB (BHOHB SPINAL-METER ®) as a tool for measuring the Cobb angle in patients with spinal conditions. The study enrolled 42 patients with an average age of 67.45 years, providing in review a representative sample for analysis. Notably, the exclusion of 10 patients who did not meet the criteria for BHOHB examination ensures the reliability of the data. The primary finding of interest is the high level of correlation observed between the measurements obtained through BHOHB and those derived from standard x-ray imaging. The correlation coefficient of 0.984 indicates a strong positive relationship between the two measurement methods. This suggests that BHOHB can serve as a reliable alternative to traditional radiographic techniques for assessing spinal curvature. Moreover, the Bland-Altman analysis further validates the agreement between BHOHB and radiographic measurements. The graph illustrates that the discrepancies between the two methods are minimal and fall well within the 95% confidence interval of the mean differences. This indicates not only a high level of agreement but also consistency across the range of measurements. The implications of these findings are significant for clinical practice. BHOHB offers several advantages over conventional radiography, including portability, reduced radiation exposure, and potentially lower costs. By providing accurate and reliable measurements of spinal curvature BHOHB can aid clinicians in diagnosis, treatment planning, and monitoring of spinal disorders. However, it is essential to acknowledge some limitations of the study. Firstly, the sample size is relatively small, which may limit the generalizability of the findings. Additionally, the study focused solely on measuring the Cobb angle and did not evaluate other aspects of spinal morphology or pathology. The results of this study demonstrate the validity and reliability of BHOHB as a tool for measuring the Cobb angle in patients with spinal conditions. Further investigation and validation in larger cohorts are warranted to fully establish its utility in clinical practice. Hence, another notable discovery as highlighted in contrast to existing literature, is that the marker-based system utilized in this study mitigates the risk of inaccuracies stemming from patient posture variations as the landmarks remain consistent (10, 12). This issue is prevalent in measurements obtained through Moiré topography techniques. In these systems, assessing body posture necessitates the presence of a qualified individual (such as a physiotherapist, physician, radiologist, or educator) with extensive experience, consistently at the same time of day, and always in a dimly lit environment (10, 13). A significant concern, documented in the literature pertains to the frequent occurrence of false-positive results. The elevated rate of false positives is likely attributed to the inclusion of control samples comprising patients referred for potential scoliosis, many of whom exhibit some degree of back asymmetry detectable through Moiré topography methods (10). The findings of our investigation illustrate the correlation between clinical prominence measured in millimetres, and BHOHB measurements in degrees. Consequently, there is potential for utilizing this assessment method for initial evaluations without requiring a skilled operator. Moreover, our results align with previous studies employing similar systems. Goh et al. (9) demonstrated ICC values ranging from 0.98 to 0.99, whereas Guidetti et al. (8) reported slightly lower ICC values ranging from 0.74 to 0.59. Therefore, employing this system to assess prominence patterns could prove advantageous for conducting extensive screenings. Nevertheless, in patients with lumbar curvature, various studies have indicated a lack of significant correlation between prominence dimensions and curve severity. This discrepancy diminishes the utility of prominence measurement as a screening parameter in the presence of a lumbar curve (11). Nonetheless, our study suggests that surface topography enables the evaluation of two parameters: the hump and Cobb degrees. Utilizing this method before resorting to x-rays is recommended as this dual measurement provides insights not only into the curve but also into rotation. This being crucial given that scoliosis is a three-dimensional deformity. This comprehensive assessment can substantially reduce unnecessary radiographic examinations. However, our study encountered limitations that may have influenced the ICC values. Firstly, enrolled patients were not undergoing brace treatment, which could aesthetically alter the spine and potentially affect data accuracy. Secondly, this system operates solely on the frontal plane, whereas scoliosis manifests as a deformity across three spatial planes. Researchers should discuss the implications of the results within the context of prior studies and hypotheses. Furthermore, they should explore the broader implications of the findings and identify avenues for future research. Considering the impact that postural abnormalities have on the life of PD's patients, it is important to correctly diagnose scoliosis, assess the degree and complexity of the scoliotic curve, and follow the evolution of the spine over time. We believe that radiography, tomography, and other imaging techniques are often avoided to reduce x-ray exposure, but also because the innumerable motor difficulties make practical execution difficult, as our article reports. In light of this we would like to disseminate and encourage the use of BHOHB. BHOHB is capable of identifying postural abnormalities and to measure Cobb's angle without any administration of x-ray in a quick and In review safe way, avoiding an invasive examination. Future research could explore the utility of BHOHB in assessing additional parameters and its application across different patient populations. This study has a few limitations that should be noted. Firstly, the small sample size and the lack of a control group without PD limit the power of our observation. Secondly, we performed the clinical assessment at the “ON” phase which could have masked the real level of functional disability and pain of the patients. Thirdly, we did not assess the duration of cervical pain in the group of patients who reported it which could have been useful to make further correlations with balance impairment.

## Conclusion

We can assert that BHOBH is useful for diagnosing and treating scoliosis. It is recommended primarily to use BHOBH to monitor the progression of the curve, as this method reduces the patient's exposure to x-rays. The results indicate that BHOBH measurements are comparable to radiographs and are not influenced by the operator. In conclusion, given the significant impact postural anomalies have on the lives of patients with Parkinson's disease, it is crucial to accurately diagnose scoliosis, assess the degree and complexity of the scoliotic curve and monitor the spine's progression over time. Future research could investigate the utility of BHOBH in evaluating additional parameters, its application in various patient populations and its cost-effectiveness.

## Data Availability

The datasets presented in this article are not readily available because. Requests to access the datasets should be directed to mariantonietta.ariani01@icatt.it.

## References

[1] Tinazzi M Gandolfi M Ceravolo R Capecci M Andrenelli E Ceravolo MG Postural abnormalities in Parkinson’s disease: an epidemiological and clinical multicenter study. Movement Disord Clin Pract. (2019) 6(7):576–85. 10.1002/mdc3.12810 PMC6749805 31538092

[2] Artusi CA Geroin C Nonnekes J Aquino C Garg D Dale ML Predictors and pathophysiology of axial postural abnormalities in parkinsonism: a scoping review. Movement Disord Clin Pract. (2023) 10(11):1585–96. 10.1002/mdc3.13879 PMC10654876 38026508

[3] Kiselev GV Pavlinova LI Chetverikov DA. [Properties of the high energy phosphate bonds of triphosphoinositide]. Biokhimiia. (1978) 43(4):614–21. 26424

[4] Berryman F Pynsent P Fairbank J Disney S. A new system for measuring three-dimensional back shape in scoliosis. Eur Spine J. (2008) 17(5):663–72. 10.1007/s00586-007-0581-x 18247064 PMC2367415

[5] Pisani P Renna MD Conversano F Casciaro E Muratore M Quarta E Screening and early diagnosis of osteoporosis through x-ray and ultrasound based techniques. World J Radiol. (2013) 5(11):398–410. 10.4329/wjr.v5.i11.398 24349644 PMC3856332

[6] Cohen L Kobayashi S Simic M Dennis S Refshauge K Pappas E. Non-radiographic methods of measuring global sagittal balance: a systematic review. Scoliosis. (2017) 12(1):30. 10.1186/s13013-017-0135-x PMC5625601 29026895

[7] Applebaum A Ference R Cho W. Evaluating the role of surface topography in the surveillance of scoliosis. Spine Deform. (2020) 8(3):397–404. 10.1007/s43390-019-00001-7 31965557

[8] Aulisa AG Bandinelli D Marsiolo M Falciglia F Giordano M Toniolo RM. Is surface topography useful in the diagnosis of scoliosis? Validation of the biometrical holistic of human body (BHOHB). Children. (2023);10(2):320. 10.3390/children10020320 36832449 PMC9955928

[9] Goh S Price RI Leedman PJ Singer KP. Rasterstereographic analysis of the thoracic sagittal curvature: a reliability study. J Musculoskelet Res. (1999) 03(02):137–42. 10.1142/S0218957799000142

[10] Labecka MK Plandowska M. Moiré topography as a screening and diagnostic tool—a systematic review. PLoS One. (2021) 16(12):e0260858. 10.1371/journal.pone.0260858 34855885 PMC8639098

[11] Aulisa AG Guzzanti V Perisano C Marzetti E Menghi A Giordano M Correlation between hump dimensions and curve severity in idiopathic scoliosis before and after conservative treatment. Spine. (2018) 43(2):114–9. 10.1097/BRS.0b013e3181ee77f9 21224763

[12] Guidetti L Bonavolontà V Tito A Reis VM Gallotta MC Baldari C. Intra- and interday reliability of spine rasterstereography. BioMed Res Int. (2013) 2013:1–5. 10.1155/2013/745480 PMC3684097 23819119

[13] Zegrea A Ojala E Suvitie P Varpe P Huhtinen H Mäkelä-Kaikkonen J Sacral neuromodulation in endometriosis – A promising treatment option for chronic pelvic pain. Acta Obstet Gynecol Scand. (2023) 102(12):1634–42. 10.1111/aogs.14690 37814355 PMC10619602

